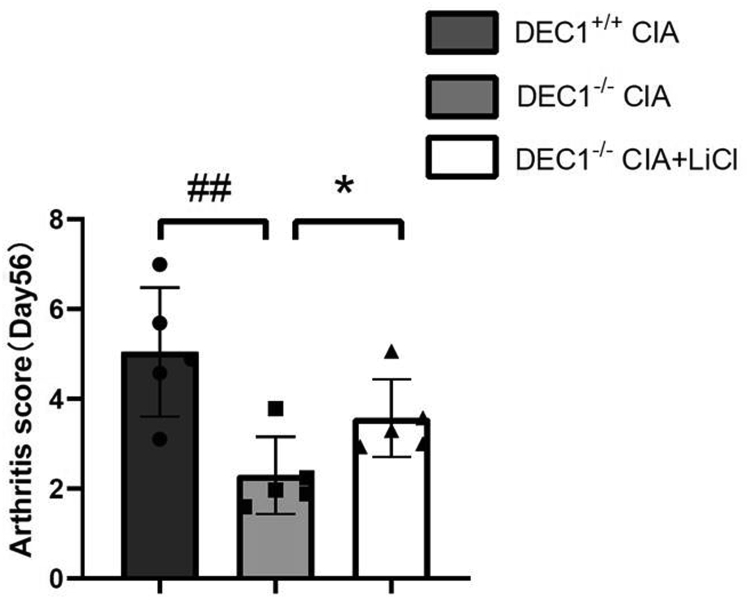# Differentiated embryonic chondrocyte expressed gene-1 is a central signaling component in the development of collagen-induced rheumatoid arthritis

**DOI:** 10.1016/j.jbc.2023.102982

**Published:** 2023-02-03

**Authors:** Yichen Wu, Haobin Wang, Ying Huo, Bingfang Yan, Hiroaki Honda, Wei Liu, Jian Yang

**Affiliations:** 1 Department of Pharmacology, Nanjing Medical University, Nanjing, China; 2 Department of Pharmacology, James L. Winkle College of Pharmacy University of Cincinnati, Cincinnati, Ohio, USA; 3 Major in Advanced Life Sciences and Medicine, Institute of Laboratory Animals, Tokyo Women's Medical University, Tokyo, Japan

**Keywords:** rheumatoid arthritis, differentiated embryonic chondrocyte expressed gene 1, PI3KCA(p110α)/Akt/GSK3β, Wnt/β-catenin and NFATc1, Akt, protein kinase B, CIA, collagen-induced arthritis, COX-2, cyclooxygenase-2, CTSK, cathepsin K, CTSL, cathepsin L, DEC1, differentiated embryo-chondrocyte expressed gene 1, DMEM, Dulbecco's modified Eagle's medium, FBS, fetal bovine serum, FLS, fibroblast-like synovial, GSK3β, glycogen synthase kinase-3β, ICAM-1, intercellular adhesion molecule 1, IHC, immunohistochemistry, IL, interleukin, JAK, Janus kinase, LiCl, lithium chloride, LPS, lipopolysaccharide, MMP, matrix metalloproteinase, NFATc1, nuclear factor of activated T-cell, OB-cadherin, osteoblast cadherin, PI3KCA, PI3K catalytic subunit alpha, RA, rheumatoid arthritis, RANKL, receptor activator of NF-κB ligand, STAT, signal transducer and activator of transcription, TNF-α, tumor necrosis factor-α

## Abstract

Rheumatoid arthritis (RA) is one of the most common autoimmune diseases and affects almost 1% of the population. Differentiated embryo-chondrocyte expressed gene-1 (DEC1) has been associated with both osteogenesis and osteoclastogenesis. RA condition is marked by inflammatory hyperplasia, and DEC1 is known to support inflammatory reactions and implicated in antiapoptosis and cell invasion. Here, our goal was to test the hypothesis that DEC1 enhances RA development induced by collagen-induced arthritis (CIA), a well-recognized protocol for developing RA animal models. DEC1^+/+^ and DEC1^−/−^ mice were subjected to CIA protocol, and the development of RA condition was monitored. We found that CIA robustly induced RA phenotypes (*e.g.*, synovial hyperplasia) and greatly increased the expression of proinflammatory cytokines such as TNF-α. However, these changes were detected in DEC1^+/+^ but not DEC1^−/−^ mice. Interestingly, these very cytokines strongly induced DEC1, and such a dual role of DEC1, as an inducer for and being induced by proinflammatory cytokines, constitutes a DEC1-amplifying circuit for inflammation. Knockdown of DEC1 in human MH7A cells strongly decreased cell migration and invasion as well as the expression of genes related to RA phenotypes. The combination of DEC1-directed migration and invasion *in vitro* with synovial hyperplasia *in vivo* mechanistically establishes cellular bases on how DEC1 is involved in the development of RA phenotypes. In addition to inflammatory signaling, DEC1 functionally interacted with PI3KCA(p110α)/Akt/GSK3β, Wnt/β-catenin, and NFATc1. Such engagement in multiple signaling pathways suggests that DEC1 plays coordinated and integral roles in developing RA, one of the most common autoimmune diseases.

Rheumatoid arthritis (RA) is a chronic systematic autoimmune disease characterized by symmetrical inflammatory polyarthritis (1). RA has several notable symptoms, including joint swelling, pain, stiffness, and functional disability (2). Some of the symptoms such as stiffness are worse in the morning (2), pointing to an involvement of circadian rhythms of inflammatory cytokines such as tumor necrosis factor-α (TNF-α) and interleukin-6 (IL-6) in RA (3, 4). The inflammatory processes in the affected joints are often accompanied by neovascularization and synoviocyte hyperplasia (*i.e.*, pannus) as well as cartilage and bone destruction (4, 5). Inflammatory cytokines activate osteoclasts, causing bone destruction in RA (6). In addition, excessive proliferation of synoviocytes enhances cellular invasion and leads to bone deformation (7). Therefore, the affected joints in RA are pathologically featured by increased synovial inflammation, pannus formation, and progressive cartilage destruction and bone resorption (8).

Multiple signaling pathways are reportedly involved in the development and progress of RA condition (9). Although these pathways exert broad activities, some are predominately involved in inflammation but others in cell proliferation. The NF-κB pathway, for example, is known to support inflammatory response, and excessive activation of NF- κB leads to abnormal fibroblast-like synovial (FLS) cell apoptosis (9, 10), a critical event for the development of RA condition. Likewise, the Janus kinase/signal transducer and activator of transcription (JAK/STAT) pathway, a well-established signaling cascade (9, 11), is intimately involved in the progression of RA condition (11). Specifically, proinflammatory cytokines such as TNF-α trigger the phosphorylation of JAK and activate the STAT proteins (11). Activation of STATs is related to persistent inflammation and the severity of RA joint destruction (11). In addition, activation of the JAK/STAT signaling has been linked to cartilage proliferation and erosion in RA synovium (9, 11, 12).

In contrast to the NF- κB and JAK/STAT pathways predominantly involved in inflammatory response, the PI3K catalytic subunit alpha/protein kinase B (PI3KCA/Akt) pathway contributes significantly to the amplification phase of RA such as abnormal proliferation of FLS cells and the differentiation of osteoclasts (13, 14). The PI3KCA(p110α)/Akt pathway is established to coordinate several signaling proteins (14). Akt is activated by phosphorylation, and the phosphorylated Akt in turn phosphorylates GSK3β (glycogen synthase kinase-3β) (13). GSK3β is a common protein shared by and mediates crosstalk between PI3K/Akt and Wnt/β-catenin pathways (15). It has been reported that Wnt/β-catenin regulates synovial inflammation, apoptosis, and proliferation as well as the expression of fibronectin and metalloproteinases in FLS (16). As seen with β-catenin, inhibition of GSK3β leads to increased transactivation activity of NFATc1 (nuclear factor of activated T-cells) (17). NFATc1 is known to regulate osteoclast functionality including osteoclast differentiation (18) and the expression of effector genes in these cells such as cathepsin K (CTSK) and matrix metalloproteinase-9 (MMP9).

Differentiated embryo-chondrocyte expressed gene 1 (DEC1) belongs to the basic helix–loop–helix family and is a potent regulator of several cellular events, including proliferation, differentiation, apoptosis, and invasion (19, 20, 21, 22, 23). This transcription factor is intimately involved in an array of pathophysiologic processes, such as circadian rhythms, lymphoid maturation, metabolic homeostasis, oncogenesis, and skeletal development (24, 25, 26, 27). Indeed, DEC1^−/−^ mice at an age of 4 weeks, compared with the age-matched WT littermates, exhibit retarded bone (28). The expression of DEC1 is significantly increased in inflammatory conditions such as human chronic gingivitis (29). Likewise, many proinflammatory cytokines such as TNF-α potently induce DEC1. Importantly, DEC1 has been functionally linked to the PI3KCA/Akt, Wnt/β-catenin, and JAK/STAT pathways (28, 30, 31).

The aim of this study was to test the hypothesis that DEC1 enhances RA development and progress induced by collagen-induced arthritis (CIA), a well-recognized protocol for developing RA animal models (32). DEC1^+/+^ and DEC1^−/−^ mice were subjected to CIA protocol and monitored for changes of synovial inflammation, pannus information, and cartilage/bone destruction. The expression of β-catenin and NFATc1 as well as the signaling activities of PI3KCA/Akt/GSK3β were determined. As expected, DEC1^+/+^ CIA mice developed strong RA symptoms, such as joint redness, swelling, and rigidity. In contrast, DEC1^−/−^ CIA mice exhibited little abnormality. Knockdown of DEC1 in human RA synovial fibroblast cell line (MH7A) significantly decreased cell migration and invasion as well as RA-related effector genes such as COX-2 (cyclooxygenase-2), OB-cadherin (osteoblast cadherin), and ICAM-1 (intercellular adhesion molecule 1). These *in vivo* and *in vitro* findings have concluded that DEC1 is an RA enhancer, and DEC1 deficiency protects against the development and progress of RA condition.

## Results

### Increased expression of DEC1 in knee joint and synovium under RA condition

DEC1 has been linked to bone metabolism (28, 31). We first tested whether the expression of DEC1 is altered under RA condition. Mice were subjected to CIA induction, and the expression of DEC1 was determined. Mice received one injection on day 0 and booster injection on day 21 (Fig. S2*A*). On day 56, mice were euthanized and samples were collected to analyze clinical presentation and gene expression. Clinically, CIA-induction mice developed strong RA clinical phenotypes, such as redness and swelling in the knees, ankles, and hind paws (Fig. S2*D*). Proteins were extracted from mice knee joints and significant increases (greater than twofold) of DEC1 in CIA mice compared with the controls (Fig. S2*B*). Immunohistochemistry (IHC) staining specified the increase in DEC1 primarily located in the synovial tissues, especially in the hyperplasic synovium and pannus of CIA mice (Fig. S2*C*).

### Development of RA phenotypes as a function of DEC1 genotype (DEC1^+/+^ or DEC1^−/−^)

**Figure 1:**
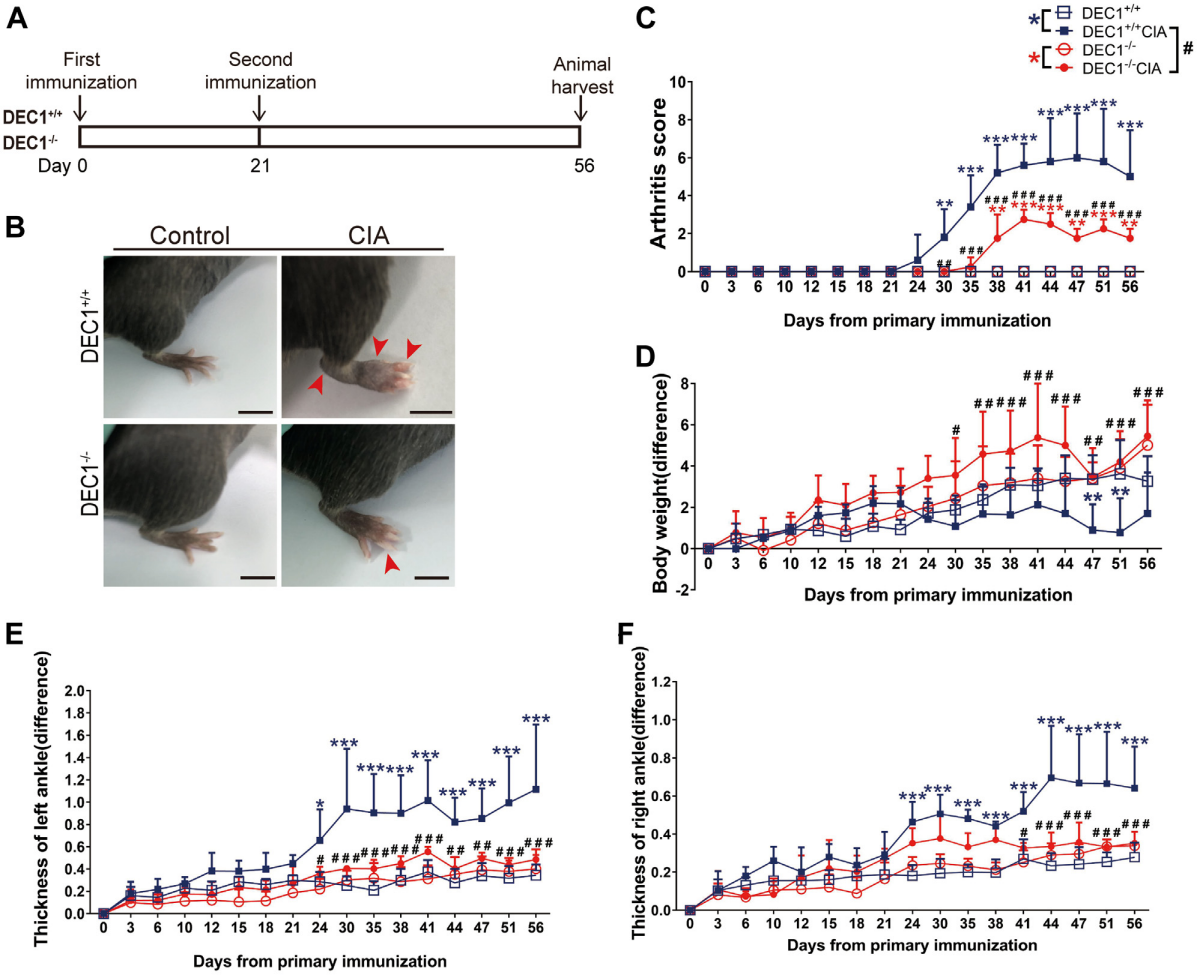
**Development of RA phenotypes as a function of DEC1 genotype.***A*, the flow chart of establishing RA model induced by CIA in DEC1^+/+^ and DEC1^−/−^ mice. *B*, representative images of ankle joint from DEC1^+/+^ and DEC1^−/−^ CIA mice on day 56. Scale bar represents 5 mm. *C*, arthritis score (AS) of ankle joint from DEC1^+/+^ and DEC1^−/−^ CIA mice. *D*, body weight of DEC1^+/+^ and DEC1^−/−^ CIA mice. *E*, thickness of left ankle joint from DEC1^+/+^ and DEC1^−/−^ CIA mice. *F*, thickness of right ankle joint from DEC1^+/+^ and DEC1^−/−^ CIA mice. The data are expressed as mean ± SD (n = 5 mice in each group) with statistical significance made at the level of ∗*p* < 0.05, ∗∗*p* < 0.01, or ∗∗∗*p* < 0.001 between CIA mice (DEC1^+/+^ or DEC1^−/−^) and their corresponding controls. Statistical significance was also made at the level of ^#^*p* < 0.05, ^##^*p* < 0.01, or ^###^*p* < 0.001 between DEC1^+/+^ and DEC1^−/−^ CIA mice. CIA, collagen-induced arthritis; DEC1, differentiated embryo-chondrocyte expressed gene 1; RA, rheumatoid arthritis.

The increased expression of DEC1 in CIA mice suggested that DEC1 plays a role in RA development. To shed light on this possibility, DEC1^+/+^ and DEC1^−/−^ mice were subjected to RA induction by CIA (Fig. 1*A*), and the development of RA phenotypes was monitored, including body weight, thickness of ankles, and arthritis score. As expected, DEC1^+/+^ CIA mice but not DEC1^+/+^ control mice (no CIA induction) had joint redness, swelling, and rigidity (*e.g.*, hind paws) (*upper* of Fig. 1*B*). In contrast, these changes were much reduced in DEC1^−/−^ CIA mice (*bottom* of Fig. 1*B*). Likewise, DEC1^−/−^ CIA mice showed significantly reduced severity in terms of arthritis scores by greater than twofold (Fig. 1*C*), thickness of ankles, and weight loss after the second immunization was administered (Fig. 1, *D*–*F*). These findings conclude that DEC1 is an enhancer for RA development.

### Protection of DEC1 deficiency against joint erosion and deformation

**Figure 2:**
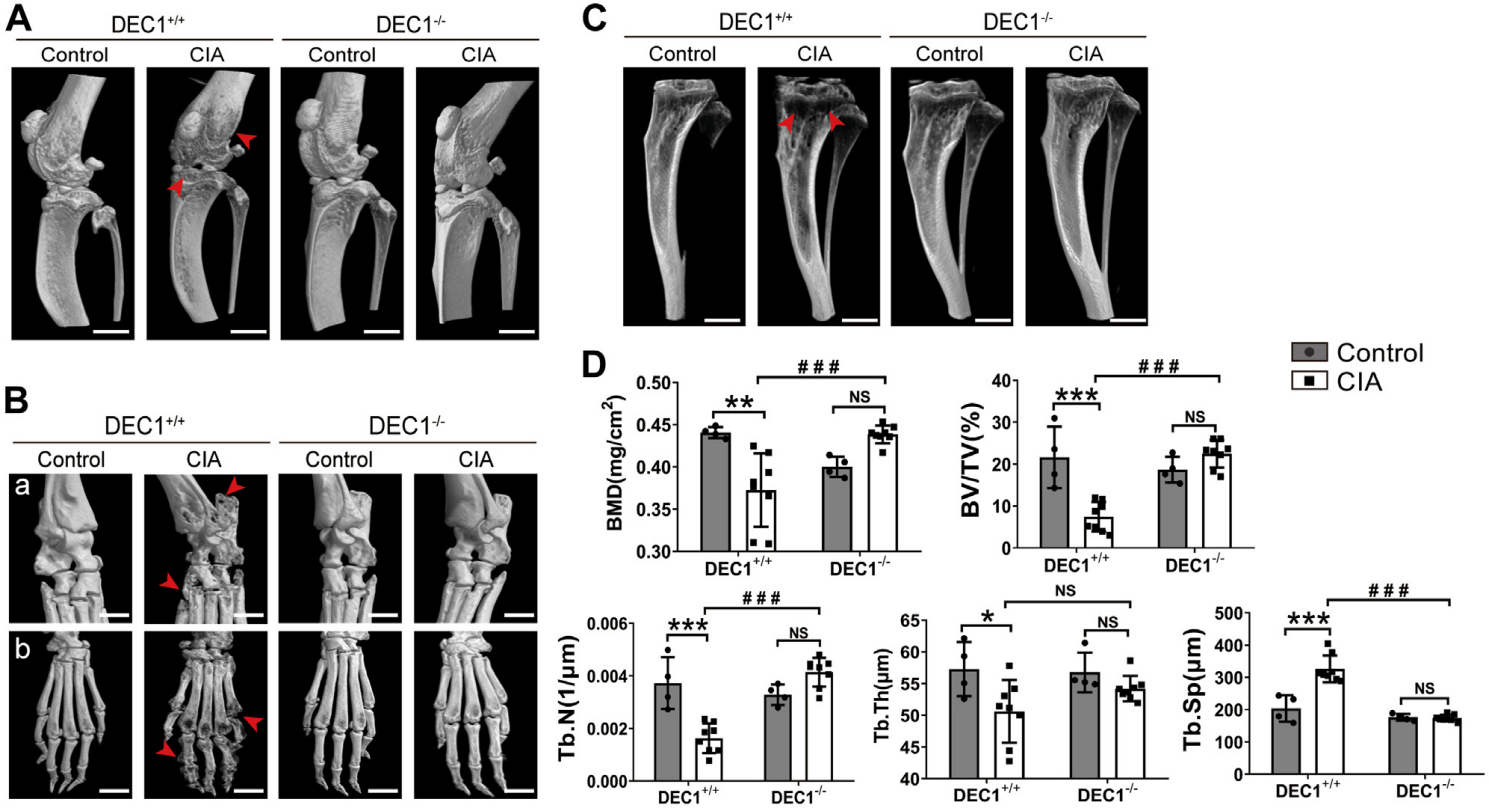
**Protection of DEC1 deficiency against joint erosion and deformation.** Micro-CT scanning and three-dimensional reconstruction of knee (*A*), ankle and hind paw (*B*), and tibia (*C*) between DEC1^+/+^and DEC1^−/−^ CIA mice. *D*, quantification of bone mineral density (BMD), trabecular bone volume to total volume fraction (BV/TV), trabecular number (Tb.N), the separation between individual trabecular (Tb.Sp), and trabecular thickness (Tb.Th) of proximal tibia from DEC1^+/+^ and DEC1^−/−^ CIA mice by micro-CT. The data are expressed as mean ± SD (n = 5 mice in each group) with statistical significance made at the level of ∗*p* < 0.05, ∗∗*p* < 0.01, or ∗∗∗*p* <0.001 between CIA mice (DEC1^+/+^ or DEC1^−/−^) and their corresponding controls. Statistical significance was also made at the level of ^###^*p* < 0.001 between DEC1^+/+^ CIA and DEC1^−/−^ CIA mice. Scale bar represents 2 mm. Data are analyzed using two-way ANOVA; differences between groups are analyzed using *t* test. CIA, collagen-induced arthritis; DEC1, differentiated embryo-chondrocyte expressed gene 1.

Joint erosion and deformation are key features of RA (4, 5, 33). We next investigated the changes of the joint inner structures by micro-CT scanning and histological staining. Compared with DEC1^+/+^ control mice, DEC1^+/+^CIA mice showed severe erosion, deformation of knee and ankle joints, and tibia (Fig. 2, *A*–*C*). In contrast, DEC1^−/−^ CIA mice showed much less severity in these changes compared with their DEC1^+/+^ counterparts. Furthermore, micro-CT scanning showed significantly reduced trabecula number and density in DEC1^−/−^ CIA mice compared with DEC1^+/+^ CIA mice in relation to their respective controls.

Quantitatively, DEC1^+/+^ CIA mice, compared with DEC1^+/+^ control mice, showed 10 to 60% decreases in bone mineral density, trabecular bone volume to total volume fraction, Tb.N, and Tb.Th. In contrast, Tb.Sp increased by ∼50% in DEC1^+/+^ CIA over DEC1^+/+^ control mice. Between DEC1^−/−^ CIA and DEC1^−/−^ control mice, these changes were largely reversed, but none of the changes reached the level of statistical significance. Furthermore, DEC1^−/−^ CIA mice, compared with their DEC1^+/+^ counterparts, showed significant increases in all parameters examined with exceptions of Tb.Sp and Tb.Th (Fig. 2*D*). Tb.Sp was significantly decreased in DEC1^−/−^ CIA mice, whereas Tb.Th was increased, but the increase did not reach the level of statistical significance (Fig. 2*D*).

### Inhibited inflammatory hyperplasia of synovium in DEC1^−/−^ mice

**Figure 3:**
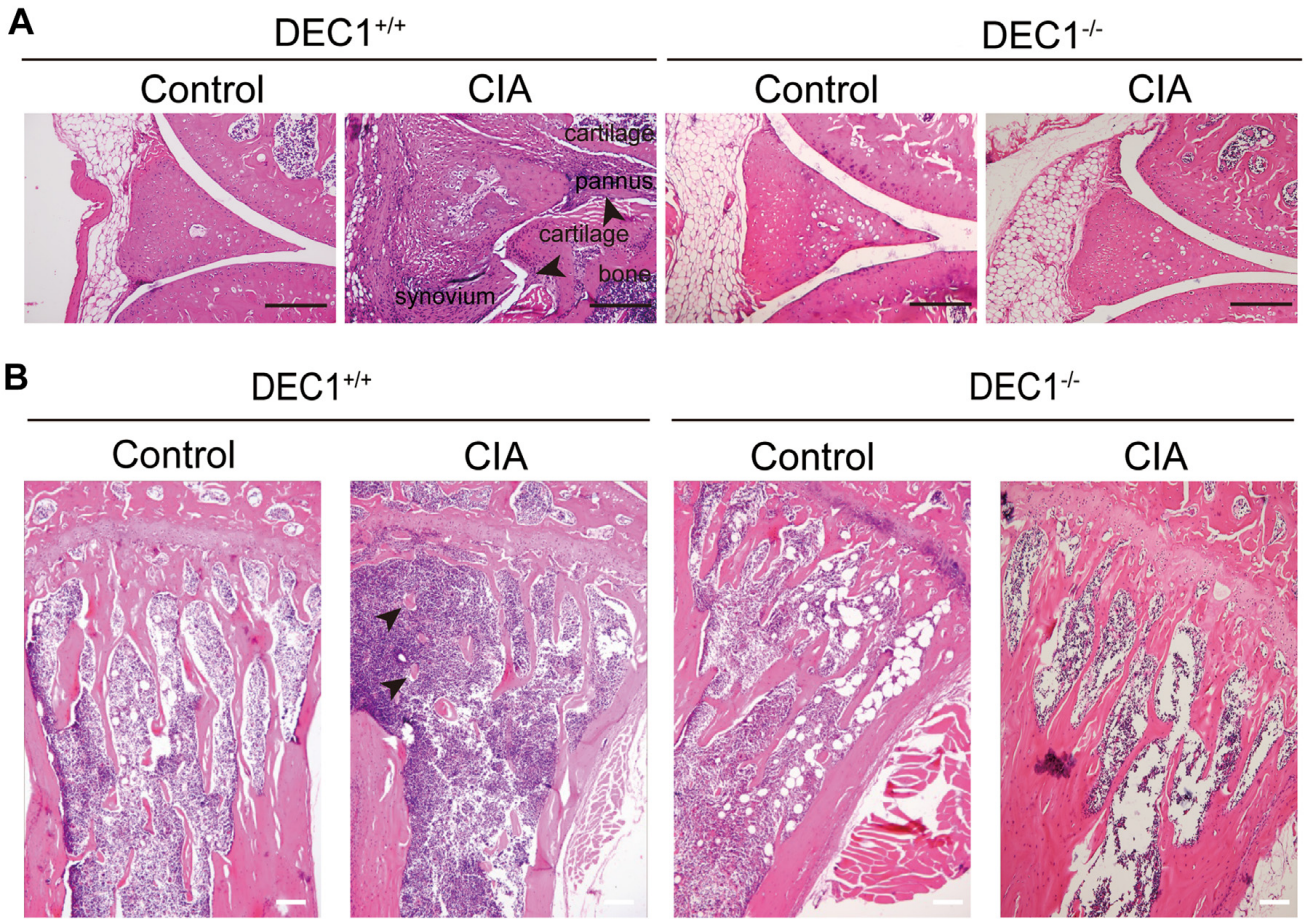
**Inhibited hyperplasia of synovium in DEC1**^**−/−**^**mice.***A*, H&E staining of knee joint (synovium) from DEC1^+/+^ and DEC1^−/−^ CIA mice (n = 3 mice in each group). The *upper arrow* shows the invasive pannus extending to the articular surface and attaching the cartilage in DEC1^+/+^ CIA mice. The *lower arrow* shows the cartilage and bone erosion by hyperplasic synovium in DEC1^+/+^ CIA mice. *B*, H&E staining of proximal tibia from DEC1^+/+^ and DEC1^−/−^ CIA mice (n = 3 mice in each group). Scale bar represents 100 μm. CIA, collagen-induced arthritis; DEC1, differentiated embryo-chondrocyte expressed gene 1.

Inflammatory hyperplasia of synovium is one of the top serious pathological changes throughout RA initiation and development (4, 5, 33). We next tested whether DEC1 regulates events closely related to such hyperplasia, including synovial inflammation, pannus formation, cartilage, and bone destruction in joints. As shown in Figure 3*A* (*left*), DEC1^+/+^ CIA mice, compared with DEC1^+/+^ control mice, showed increases of the proliferation in the synovial lining with enhanced formation of synovial pannus. In addition, the cavity of the knee was almost invisible in DEC1^+/+^ CIA mice compared with the control mice (*left* of Fig. 3*A*). In contrast, the synovial lining and knee cavity were comparable between DEC1^−/−^ CIA and control mice (*right* of Fig. 3*A*). In addition, DEC1^−/−^ CIA mice showed healthier and more complete trabecular compared with DEC1^+/+^ CIA mice (Fig. 3*B*). Clearly, DEC1 deficiency protected against CIA-induced synovial hyperplasia/erosion and enhanced pannus formation (Fig. 3).

To gain cellular and molecular understanding, we next determined the expression of genes in the knee synovium that are relevant to inflammatory response and cell migration such as COX-2, OB-cadherin, and ICAM-1 (Fig. S3*A*). The expression of these genes, as demonstrated by IHC staining, was all increased robustly among DEC1^+/+^ CIA mice compared with DEC1^+/+^ control mice (*left* of Fig. S3*A* as DEC1^+/+^ columns). In contrast, negligible differences were detected between DEC1^−/−^ CIA and the corresponding control mice (*right* of Fig. S3*A* as DEC1^−/−^ columns). Clearly, the expression of these genes was much lower in DEC1^−/−^ CIA mice than their DEC1^+/+^ counterparts (Fig. S3*A*). As summarized in Fig. S3*B*, similar changes were detected on the expression of proinflammatory cytokines granulocyte-macrophage colony-stimulating factor, IL-6, IL-17, and TNF-α. These findings support the notion that DEC1 deficiency attenuated inflammatory reactivity, the critical events in the development of RA condition.

### Reduced cartilage and bone destruction in DEC1^−/−^ CIA mice

**Figure 4:**
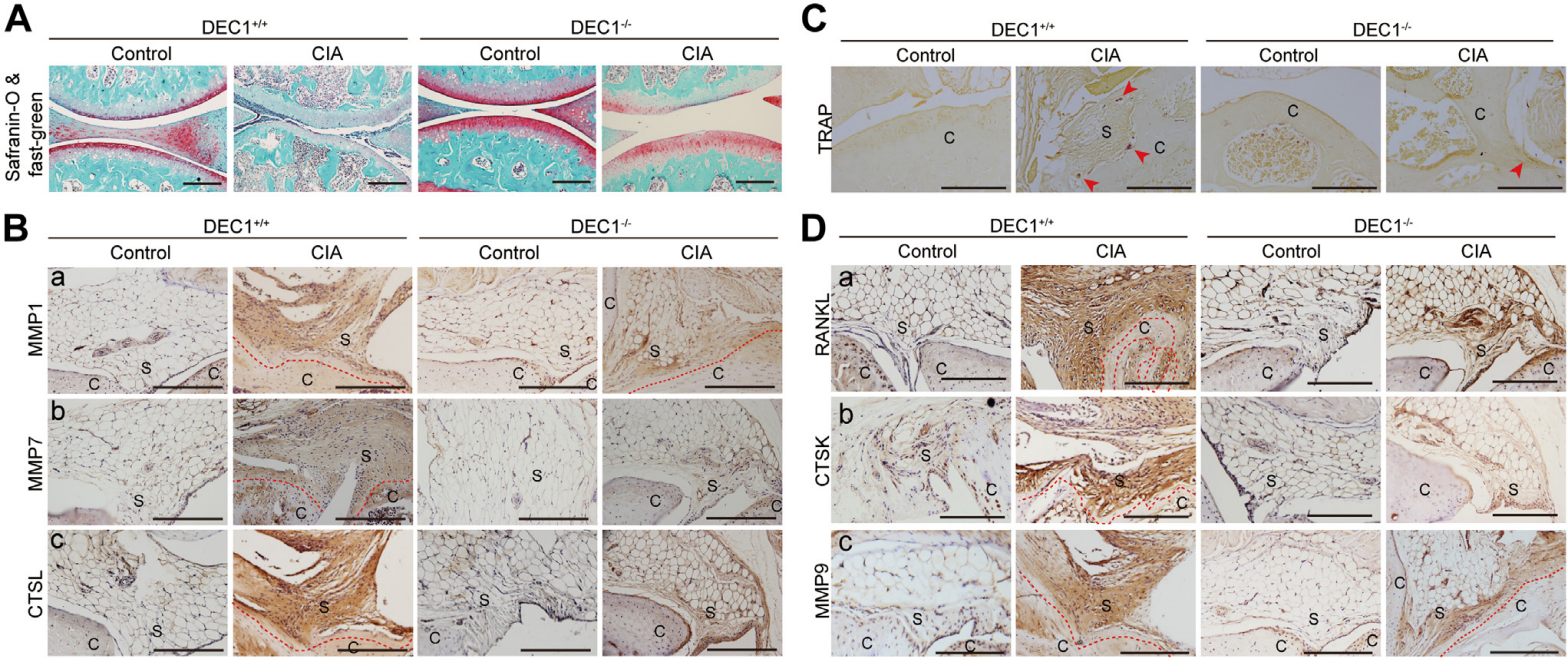
**Association of cartilage and bone destruction with DEC1 genotypes.***A*, Safranine-O and Fast Green staining of cartilage of knee joint from DEC1^+/+^ and DEC1^−/−^ CIA mice (n = 3 mice in each group). *Red staining* shows cartilage, wheraes *green staining* shows bone. *B*, MMP1, MMP7, and CTSL expression in the knee joint (synovium) from these two types of CIA mice by IHC staining (n = 3 mice in each group). *C*, TRAP staining of synovium and its adjacent joint. *D*, IHC staining of RANKL, CTSK, and MMP9 expression in synovium (n = 3 mice in each group). Scale bar represents 100 μm. C, cartilage; CIA, collagen-induced arthritis; CTSK, cathepsin K; CTSL, cathepsin L; DEC1, differentiated embryo-chondrocyte expressed gene 1; IHC, immunohistochemistry; MMP, matrix metalloproteinase; RANKL, receptor activator of NF-κB ligand; S, synovium.

Joint stiffness is a hallmark of RA condition and highly related to dysfunctional cartilage. We next tested whether DEC1 deficiency protects against cartilage damage and bone destruction in CIA mice summarized in Figure 4. The cartilage was clearly damaged in DEC1^+/+^ CIA mice but not in DEC1^+/+^ control mice based on safranine-O and fast green staining (*left* of Fig. 4*A*). In contrast, the damage of the cartilage in DEC1^−/−^ CIA mice was much less severe compared with that in DEC1^+/+^ CIA mice (*right* of Fig. 4*A*). In addition, DEC1^+/+^ CIA mice had a number of osteoclasts on the adhesion sites of the synovium (Fig. 4*C*). In the control group, the number of osteoclasts was much fewer, and the osteoclasts were principally located around the trabecular (Fig. 4*C*). Moreover, the number of tartrate-resistant acid phosphatase positive cells increased in DEC1^+/+^ CIA mice compared with that in DEC1^+/+^ control mice (Fig. 4*C*). As expected, the number of tartrate-resistant acid phosphatase positive cells in DEC1^−/−^ CIA mice were much less than that in DEC1^+/+^ CIA mice (Fig. 4*C*).

Many proteases such as MMPs participate in osteoclastic activity. Next, we tested whether RA condition increases the presence of these proteases and whether DEC1 deficiency protects against the increase. As shown in Figure 4*B* (*left*), DEC1^+/+^ CIA mice, compared with DEC1^+/+^ control mice, significantly increased the presence of MMP1, MMP7, and cathepsin L (CTSL) in the synovium and pannus based on IHC staining. In contrast, the increase of these proteases was much less in DEC1^−/−^ CIA mice (*right* of Fig. 4*B*). Similarly, DEC1^+/+^ CIA mice, compared with DEC1^+/+^ control mice, significantly increased the expression of RANKL (receptor activator of NF- κB ligand), CTSK, and MMP9 in the synovium and pannus (*left* of Fig. 4*D*), and the increase was much less in DEC1^−/−^ CIA mice than that in DEC1^+/+^ CIA mice (*right* of Fig. 4*D*).

### Involvement of DEC1 in the PI3K/β-catenin/NFATc1 signaling under RA condition

**Figure 5:**
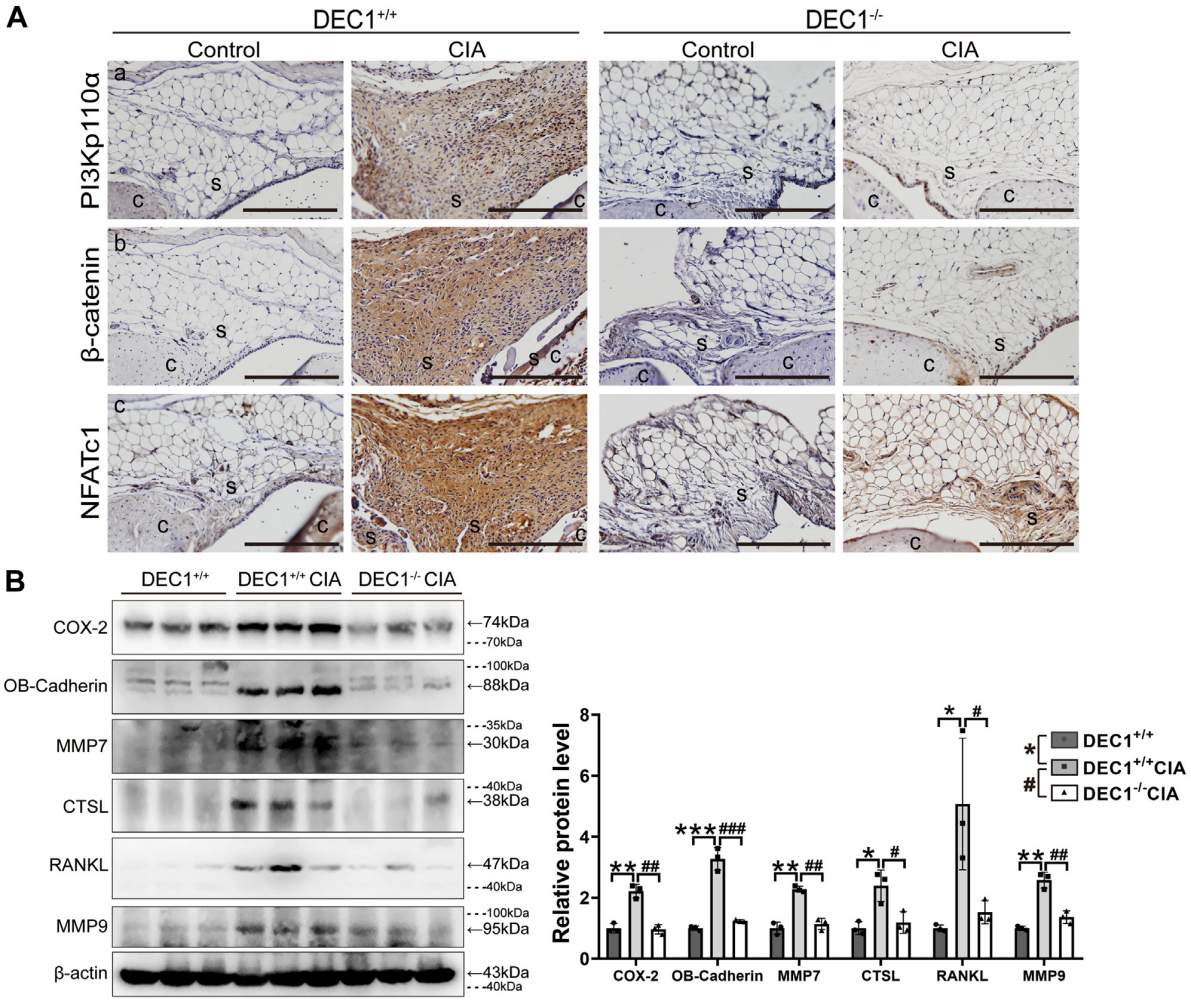
**DEC1 in the PI3K/β-catenin/NFATc1 signaling under RA condition.***A*, PI3Kp110α, β-catenin, and NFATc1 expression in the knee joint (synovium) from DEC1^+/+^ and DEC1^−/−^ CIA mice by IHC staining (n = 3 mice in each group). Scale bar represents 100 μm. *B*, COX-2, OB-Cadherin, MMP7, CTSL, RANKL, and MMP9 expression in the whole knee joint from DEC1^+/+^ control mice, DEC1^+/+^ and DEC1^−/−^ CIA mice by Western blotting. Data are expressed as mean ± SD (n = 3 mice in each group), and statistical significance was made at the level of ∗*p* < 0.05, ∗∗*p* < 0.01, and ∗∗∗ *p* < 0.001 between DEC1^+/+^ CIA and control mice. *Dashed lines* indicate representative values of ladders. Statistical significance was also made at the level of ^#^*p* < 0.05 and ^###^*p* < 0.001 between DEC1^−/−^ and DEC1^+/+^ CIA mice. Data are analyzed using two-way ANOVA; differences between groups are analyzed using *t* test. CIA, collagen-induced arthritis; C, cartilage; COX-2, cyclooxygenase-2; CTSL, cathepsin L; DEC1, differentiated embryo-chondrocyte expressed gene 1; IHC, immunohistochemistry; MMP, matrix metalloproteinase; OB-cadherin, osteoblast cadherin; RA, rheumatoid arthritis; RANKL, receptor activator of NF-κB ligand; S, synovium.

PI3KCA/Akt, Wnt/β-catenin, and NFATc1 have been established to play important roles in the development of RA (34, 35, 36). DEC1 has been shown to regulate the expression and/or signaling activity of some of these molecules (28). We next tested whether DEC1 deficiency protects against CIA induction with a possible involvement of downregulation of these genes. As shown in Figure 5*A* (*left*), DEC1^+/+^ CIA mice, compared with DEC1^+/+^ controls, robustly increased IHC staining of PI3KCA, β-catenin, and NFATc1 expression in the synovium. In contrast, the increase was negligible in DEC1^−/−^ CIA mice compared with the corresponding controls (*right* of Fig. 5*A*). To provide complementary information, Western blotting was performed for the expression of inflammatory and protease genes that are regulated by PI3KCA/Akt, Wnt/β-catenin, and/or NFATc1. As shown in Figure 5*B*, DEC1^+/+^ CIA mice significantly increased the expression of COX-2, OB-cadherin, MMP7, CTSL, RANKL, and MMP9 compared with DEC1^+/+^ control mice. In contrast, the expression of these genes was comparable between DEC1^+/+^ control mice and DEC1^−/−^ CIA mice (*right* of Fig. 5*B*).

**Figure 6:**
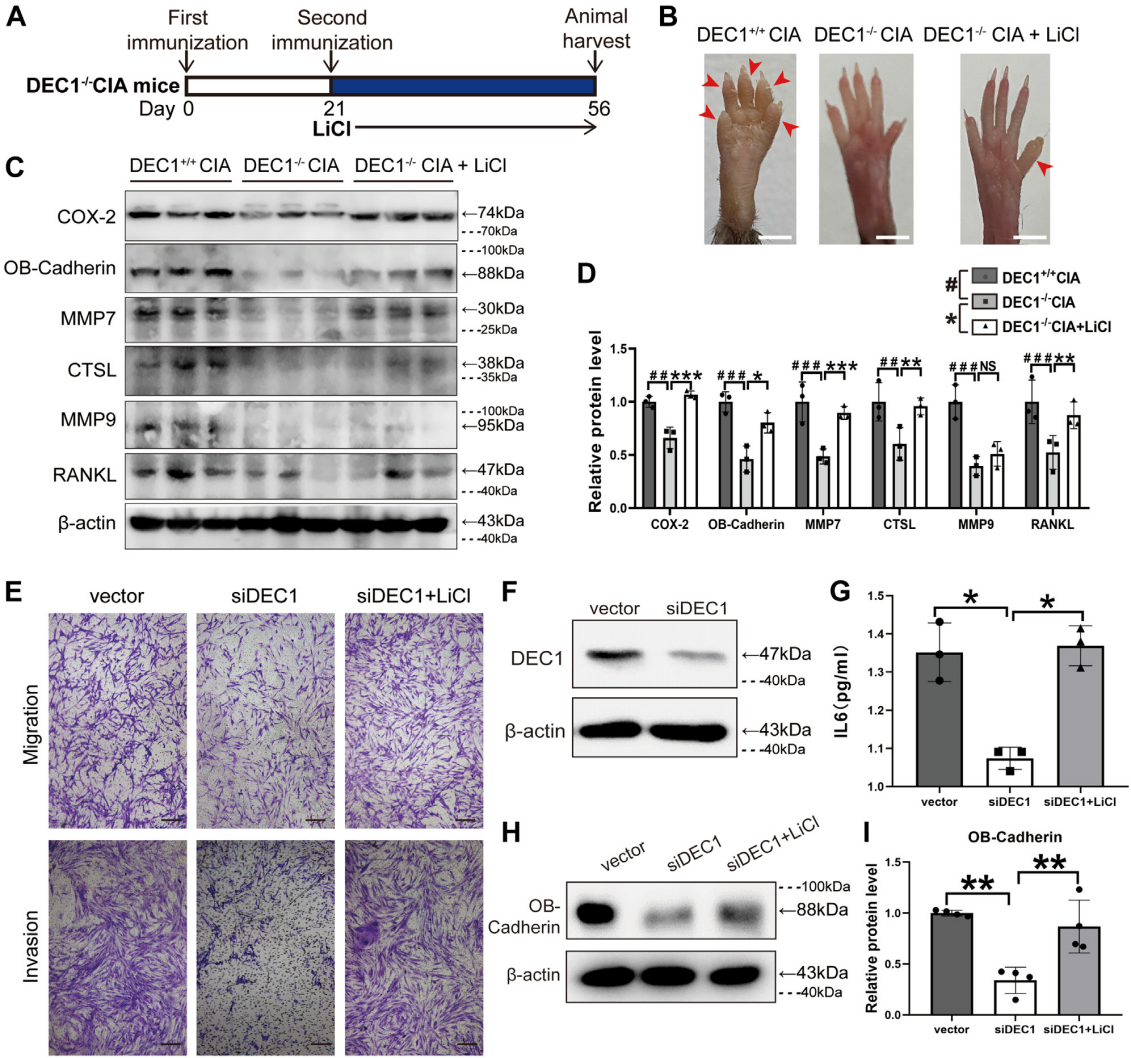
**LiCl reversed the reduction of RA phenotypes in DEC1**^**−/−**^**CIA mice.***A*, diagrammatical presentation of LiCl administration in DEC1^−/−^ CIA mice. *B*, reversed reduction of hind paw swelling in DEC1^−/−^ CIA mice by LiCl (n = 3 mice in each group). Scale bar represents 2 mm. *C* and *D*, reversed protein expression of COX-2, OB-Cadherin, MMP7, CTSL, RANKL, and MMP9 in the whole knee joint in DEC1^−/−^ CIA mice by LiCl as determined with Western blotting. The data were expressed as mean ± SD (n = 3 mice in each group). Statistical significance was made at the level of ^##^*p* < 0.01 and ^###^*p* < 0.001 between DEC1^−/−^ and DEC1^+/+^ CIA mice. Statistical significance was made at the level of ∗*p* < 0.05, ∗∗*p* < 0.01, or ∗∗∗*p* < 0.001 between DEC1^−/−^ and LiCl-treated DEC1^−/−^ CIA mice. *E*, knockdown of DEC1 reduced migration and invasion, and LiCl reversed it in MH7A cells. Scale bar represents 100 μm. *F*, the efficiency of DEC1 knockdown in MH7A cells (*p* = 0.003). *G*, knockdown of DEC1 reduced IL-6 level, and LiCl reversed it in MH7A cells. *H* and *I*, knockdown of DEC1 reduced OB-cadherin level, and LiCl reversed it in MH7A cells. *Dashed lines* indicate representative values of ladders. ∗*p* < 0.05 and ∗∗*p* < 0.01, comparisons are shown in the figure. The data were expressed as mean ± SD (MH7A experiments were repeated at least for three times in *E*–*I*. Data are analyzed using two-way ANOVA; differences between groups are analyzed using *t* test. CIA, collagen-induced arthritis; COX-2, cyclooxygenase-2; CTSL, cathepsin L; DEC1, differentiated embryo-chondrocyte expressed gene 1; MMP, matrix metalloproteinase; OB-cadherin, osteoblast cadherin; RA, rheumatoid arthritis; RANKL, receptor activator of NF-κB ligand.

The Wnt/β-catenin pathway has been implicated in the development of RA condition, and we have shown that DEC1^+/+^ CIA but not DEC1^−/−^ CIA mice robustly increased the expression of β-catenin (Fig. 5*A*). We next tested whether lithium chloride (LiCl), an activator of β-catenin (28), attenuates the protection of DEC1 deficiency against CIA induction. Three groups (DEC1^+/+^ CIA, DEC1^−/−^ CIA, and DEC1^−/−^ CIA treated with LiCl) were subjected to RA induction with CIA. The LiCl treatment was performed by intraperitoneal injection at a dose of 200 mg/kg/day starting on day 21 (second immunization with collagen) and ending on day 56 (Fig. 6*A*). The morphological changes of joints were monitored, and the expression of pathologically related genes (*e.g.*, inflammation) was determined. As shown in Figs 6*B* and S4, DEC1 deficiency protected against the swelling and rigidity of the joints compared with WT mice, whereas LiCl partially abolished this protection. As shown in Figure 6, *C* and *D*, DEC1^−/−^ CIA mice, compared with DEC1^+/+^ CIA mice, significantly decreased the expression of COX-2, OB-cadherin, MMP7, CTSL, RANKL, and MMP9. However, the decrease was almost fully reversed by LiCl treatment with an exception of MMP9 (Fig. 6, *C* and *D*). To shed light on cellular functionality of LiCl-regulated expression in relation to DEC1, MH7A cells were transfected with siDEC1 or the vector, and transfected cells were divided into three groups (vector, siDEC1, and siDEC1 + LiCl). Cell migration and invasion were determined, and the media were collected for the level of IL-6 determination. As shown in Figure 6*E*, DEC1 knockdown robustly decreased cell migration and invasion, and the decreases were reversed by LiCl treatment. Likewise, DEC1 knockdown (Fig. 6*F*) significantly decreased the secretion of IL-6 (Fig. 6*G*) and the expression of OB-cadherin (Fig. 6, *H* and *I*), and the decreases were reversed by LiCl.

### Molecular connections of DEC1 with the PI3K/β-catenin/NFATc1 signaling

The animal study, in combination with gene expression experiments, has made a strong link of DEC1 deficiency to attenuating the PI3K/β-catenin/NFATc1 signaling in protecting against CIA-induced RA condition (Figs. 1 and 5). We next tested whether such mechanistic link can be confirmed *in vitro* with human-derived cells. MH7A cell line, employed as an RA cell model (37), was used. The goal of the proposed cell model study is twofold: (a) to test whether the microenvironment of RA alters DEC1 expression and what the pathological significance is and (b) to make direct signaling connection of DEC1 with the PI3K/β-catenin/NFATc1 pathway.

**Figure 7:**
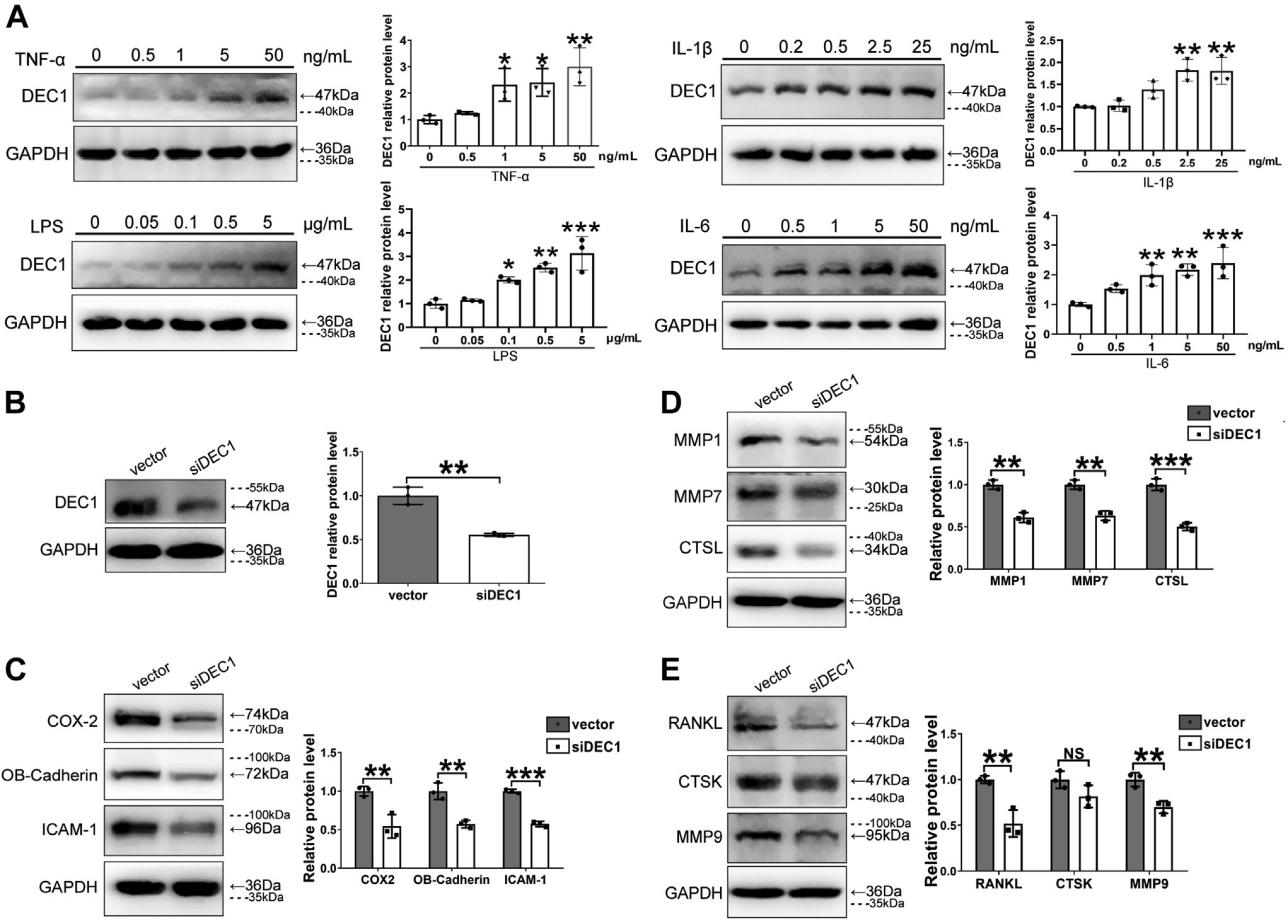
**Regulated expression of DEC1 by inflammatory factors and the expression of RA-associated genes in DEC1 knockdown cells.***A*, increased expression of DEC1 by IL-1β, IL-6, LPS, and TNF-α in MH7A cells. Data are analyzed using one-way ANOVA; differences between groups are analyzed using *t* test. *B*, the efficiency of DEC1 knockdown in MH7A cells (*p* = 0.002). *C*, downregulated expression of COX-2 (*p* = 0.009), OB-cadherin (*p* = 0.003), and ICAM-1 (*p* < 0.001) in DEC1 knockdown MH7A cells. *D*, downregulated expression of MMP1 (*p* = 0.001), MMP7 (*p* = 0.001), and CTSL (*p* < 0.001) in DEC1 knockdown MH7A cells. *E*, downregulated expression of RANKL (*p* = 0.005), CTSK (*p* = 0.107), and MMP9 (*p* = 0.006) in DEC1 knockdown MH7A cells. *Dashed lines* indicate representative values of ladders. Data were expressed as mean ± SD. All experiments were repeated at least for three times. Statistical significance was made, in comparison with the vector control, at ∗*p* < 0.05, ∗∗*p* < 0.01, ∗∗∗*p* < 0.001, and ∗∗∗∗*p* < 0.0001. NS: *p* > 0.05. COX-2, cyclooxygenase-2; CTSK, cathepsin K; CTSL, cathepsin L; DEC1, differentiated embryo-chondrocyte expressed gene 1; ICAM-1, intercellular adhesion molecule 1; IL, interleukin; LPS, lipopolysaccharide; MMP, matrix metalloproteinase; NS, not significant; OB-cadherin, osteoblast cadherin; RA, rheumatoid arthritis; RANKL, receptor activator of NF-κB ligand; TNF-α, tumor necrosis factor alpha.

To mimic the RA microenvironment, proinflammatory cytokines (TNF-α, IL-1β, and IL-6) and the immunostimulant lipopolysaccharide (LPS) were used to treat MH7A cells for 24 h. Cells were harvested, and samples were analyzed for the expression of DEC1. As shown in Figure 7*A*, all cytokines and LPS significantly induced DEC1 expression, and the induction occurred in a dose-dependent manner. To mechanistically confirm the effects of DEC1 on the development of RA phenotypes as well as gene expression, DEC1 knockdown was performed with siRNA. As shown in Figure 7*B*, siDEC1 significantly knocked down the protein expression of DEC1. As expected, the expression of RA-effector genes, including COX-2, OB-cadherin, and ICAM-1, was all decreased significantly (Fig. 7*C*). Likewise, DEC1 knockdown significantly decreased the expression of genes associated with cartilage degradation including MMP1, MMP7, and CTSL (Fig. 7*D*) as well as those associated with bone resorption such as RANKL and MMP9 with an exception of CTSK (Fig. 7*E*).

**Figure 8:**
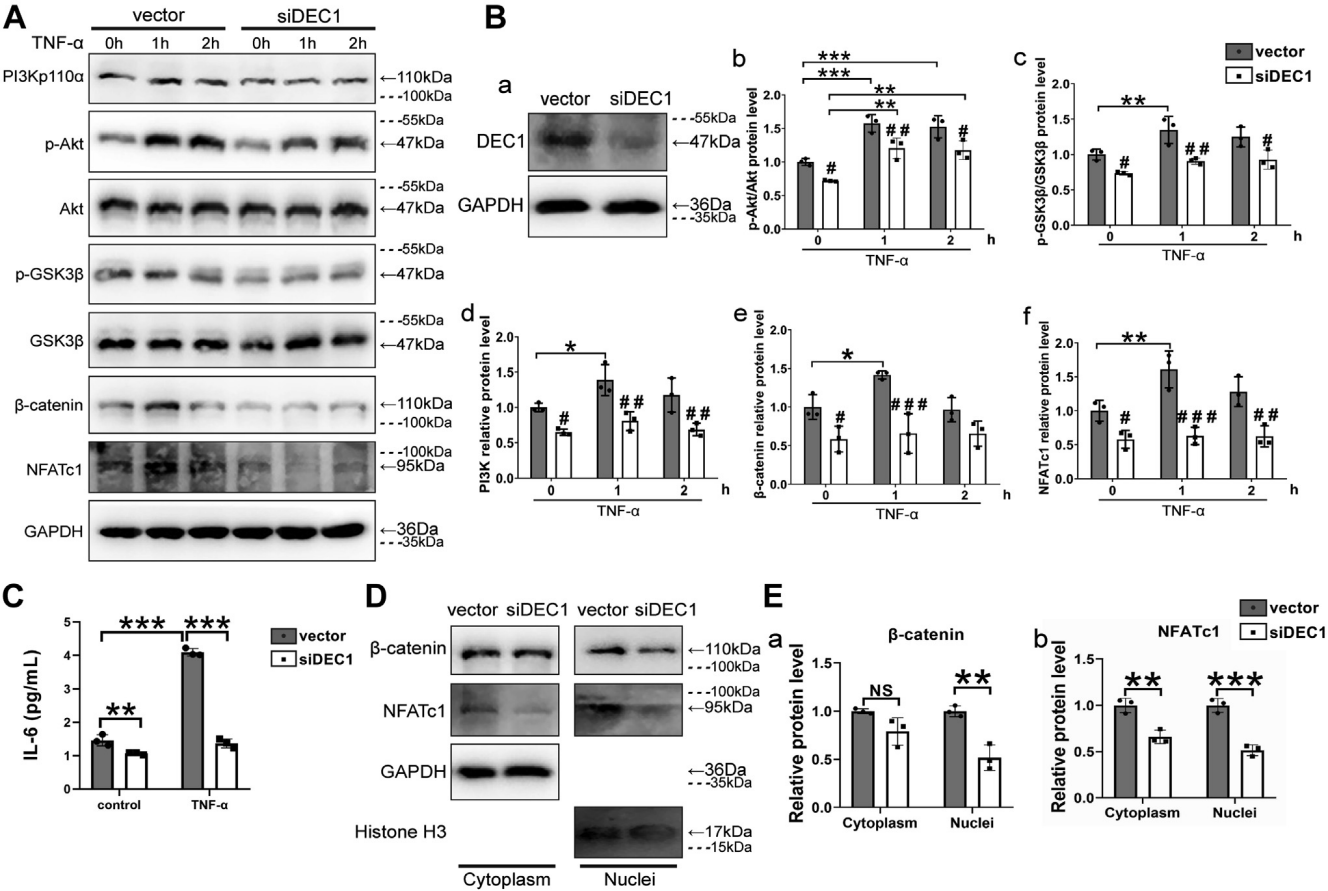
**Inhibited PI3K/β-catenin/NFATc1 signaling/expression in DEC1 knockdown cells.***A* and *B(b-f)*, decreased expression or phosphorylated ratios of PI3Kp110α, p-Akt/Akt, p-GSK3β/GSK3β, β-catenin, and NFATc1 in DEC1 knockdown MH7A cells under constitutive or cytokine-stimulated condition. The knockdown efficiency of DEC1 was also confirmed by Western blot (*B,a*). *C*, Decreased IL-6 level in DEC1 knockdown MH7A cells under TNF-α-stimulated condition. Data were expressed as mean ± SD. All experiments were repeated at least for three times. Statistical significance was made at ∗*p* < 0.05, ∗∗*p* < 0.01, and ∗∗∗*p* < 0.001 for comparisons linked by a *line*. Statistical significance was also made in comparison with the vector control at ^#^*p* < 0.05, ^##^*p* < 0.01, and ^##^*p* < 0.001 under the same treatment/time points, or comparisons are shown in the figure. Data are analyzed using two-way ANOVA; differences between groups are analyzed using *t* test. *D* and *E(a,b)*, effect of DEC1 knockdown on the cytoplasmic and nuclear presence of β-catenin (*p* = 0.066 and 0.004, respectively) and NFATc1 (*p* = 0.005 and 0.0009, respectively). The cytoplasmic and nuclear fractions from vector and siDEC1 cells were analyzed by Western blotting. *Dashed lines* indicate representative values of ladders. Data were expressed as mean ± SD. All experiments were repeated at least for three times. Statistical significance was made, in comparison with the vector control at ∗∗*p* < 0.01, ∗∗∗*p* < 0.001, and NS *p* > 0.05. DEC1, Differentiated embryo-chondrocyte expressed gene-1; GSK3β, glycogen synthase kinase-3β; IL, interleukin; NFATc1, nuclear factor of activated T-cell; NS, not significant; TNF-α, tumor necrosis factor alpha.

We next tested whether DEC1 knockdown counteracts the effect of RA environmental factors regarding the PI3K/β-catenin/NFATc1 signaling. TNF-α was used to mimic the microenvironment as this cytokine is abundantly present in the synovium and contributes significantly to RA pathogenesis (4, 5, 33). MH7A cells were transfected with the siDEC1 plasmid or the corresponding vector. The transfected cells were then treated with TNF-α (5 ng/ml) for 0 h (before treatment) to 2 h after the transfection. As shown in Figure 8, *A* and *B*, TNF-α increased the levels of PI3Kp110α (CA), p-Akt/Akt, p-GSK3β/GSK3β, β-catenin, and NFATc1 in vector-transfected MH7A cells. However, DEC1 knockdown partially or almost completely abolished such increases (Fig. 8, *A* and *B*). Likewise, TNF-α significantly increased IL-6 secretion in vector-transfected cells, and the increase was not observed in DEC1 knockdown cells (Fig. 8*C*). NFATc1 and β-catenin are transcriptional factors and require nuclear translocation to exert their activity (16, 17, 18). We next tested whether DEC1 knockdown affects their nuclear translocation. Cytoplasmic and nuclear fractions were analyzed by Western blotting. As shown in Figure 8, *D* and *E*, DEC1 knockdown significantly decreased the presence of NFATc1 and β-catenin, and the decrease was more profound in the nuclear fractions for both proteins.

## Discussion

RA is one of the most common autoimmune diseases and affects almost 1% of the population (1, 38). Pathologically, it is manifested by hyperplasic and recurrent synovial inflammation, pannus formation, cartilage degradation, and bone resorption in joints (39). The etiology of RA is multifactorial, and the underlying molecular mechanisms remain to be fully determined (1, 38). In this study, several complementary approaches have been used including *in vivo* and *in vitro* models as well as molecular and cellular experiments. We have demonstrated that CIA robustly induces RA phenotypes *(e.g.*, synovial hyperplasia) in DEC1^+/+^ mice. However, these RA phenotypical changes have been abolished partially or almost completely in DEC1^−/−^ mice (Figure 1, Figure 2, Figure 3). The *in vivo* findings have been nicely confirmed by human cell models in terms of gene expression, cellular behaviors (*e.g.*, migration), and signal integration. These findings collectively establish that DEC1 is an integral signaling component in developing RA in response to CIA induction.

CIA is widely used to induce RA condition as it closely mimics human RA pathologically and immunologically (40). Precisely, CIA induces immune response directly targeting a joint antigen (*i.e.*, collagen type II) (41). Indeed, we have shown that DEC1^+/+^ CIA mice develop synovium hyperplasia, joint swelling, cartilage degradation, and angiogenesis (Figure 1, Figure 2, Figure 3 and S2). These changes are common features in RA patients (42). Histologically, the synovium shows cellular migration and invasion; the formation of pannus is extensive and intrudes into joint cavity; and the surface of cartilage and bone attached by synovium and pannus exhibits large areas of erosion (Figs. 3 and 5, *A* and *C*). In contrast, DEC1^−/−^ CIA mice show no or minimal such changes. These observations conclude that DEC1 deficiency protects against the development of RA condition.

Consistent with the changes on RA phenotypes and histological analysis, the expression of COX-2, OB-cadherin, and ICAM-1 in the synovium is considerably increased in DEC1^+/+^ CIA mice (Fig. S3*A*). COX-2 represents the inflammation level of local tissues and is highly expressed in inflammatory synovium (43). OB-cadherin, a synoviocyte-specific adhesion molecule, promotes the migration and attachment of FLSs to the cartilage matrix and synovial lining formation (44). ICAM-1, another important adhesion molecule in RA, abundantly exists in inflammatory FLSs or vascular endothelial cells (45, 46). Likewise, the expression of inflammatory cytokines (*e.g.*, TNF-α), cartilage degradation proteases (*e.g.*, MMP7), and bone resorption proteins (*e.g.*, RANKL) is robustly increased as well in the synovium of DEC1^+/+^ CIA mice. In contrast, these changes are rather minimal in DEC1^−/−^ CIA mice (Figs. S3*B* and 4, *B* and *D*).

The *in vivo* changes of histological staining and gene expression are nicely recaptured by *in vitro* models with human-derived cells. MH7A cell line is chosen based on several important consideration: (a) this line is immortalized from human RA fibroblast-like synoviocytes (47); (b) MH7A cells are manifested as overgrowth and redistribution, two critical cellular characteristics of RA condition (33, 48); and (c) this cell line is used as an RA cell model (37). Specifically, knockdown of DEC1 decreases the expression of the inflammatory indicator COX-2 and the adhesion proteins OB-cadherin and ICAM-1 (Fig. 7*C*). Likewise, the expression of genes associated with cartilage degradation (*e.g.*, CTSL) or bone resorption (*e.g.*, MMP9) is decreased when DEC1 is knocked down (Fig. 7, *D* and *E*). Importantly, DEC1 knockdown causes robust decreases of cell migration and invasion, linking DEC1 directly to RA-associated joint deformation (Fig. 6*E*). To complement the cellular changes, proinflammatory cytokines (TNF-α, IL-1β, and IL-6) and the immunostimulant LPS, mimicking the RA joint microenvironment, all significantly induce DEC1 protein expression, and the induction occurred in a dose-dependent manner (Fig. 7*A*). Interestingly, an early study reports that TNF-α does not alter DEC1 mRNA expression (49), although this cytokine is reported to induce DEC1 in multiple human cell lines by multiple investigators (50, 51). While the precise mechanism remains to be determined, the Mrna expression, given the fact that DEC1 is a circadian gene (19), may not quite capture the time frame for traditionally function-related expression (*i.e.*, protein).

Mechanistically, our study has made several important findings linking DEC1 directly to RA signaling, particularly related to the PI3K/Akt, Wnt/β-catenin, and NFATc1 pathways (52, 53, 54). First, we have demonstrated that the level of PI3KCA protein increases significantly in the synovium of DEC1^+/+^ but not DEC1^−/−^ CIA mice (Fig. 5*A*), connecting DEC1 directly to PI3KCA/Akt signaling. Second, MH7A cells treated with TNF-α express significantly higher levels of PI3K and increase the ratios of p-Akt/Akt and Pgsk3β/GSK3β (Fig. 8, *A* and *B*). GSK3β is a common protein shared by PI3K/Akt and Wnt/β-catenin pathways (15), and increased Pgsk3β/GSK3β makes DEC1 as a functional link between these two pathways. In addition, treatment of DEC1^−/−^ CIA mice with LiCl, a known activator of the canonical Wnt/β-catenin signaling (55), reverses the improved RA phenotypes (Figs. 6*B* and S4) and the reduced expression of COX2, OB-cadherin, MMP7, CTSL, and RANKL in DEC1^−/−^ CIA mice (Fig. 6, *C* and *D*). The LiCl experiment further strengthens DEC1-mediated connection between PI3K/Akt and Wnt/β-catenin pathways in RA signaling. And finally, knockdown of DEC1 decreases β-catenin and NFATc1, and the decrease is more profound in the nuclei (Fig. 8, *D* and *E*).

The mechanisms on how DEC1 is engaged in diverse signaling pathways remain to be fully determined. Throughout these years, we have shown that DEC1 uses multiple modes of action in regulating gene expression. DEC1 is a ligand-independent transcription factor, binds to E-box or Sp1 element, and exerts transcriptionally regulatory activities (56, 57). Binding to E-box causes transcriptional repression (57), whereas binding to Sp1 site causes transactivation (57). In addition, we have shown that DEC1 interacts with other transcription factors, and the interaction delivers transactivation or transrepression activity depending on an interactive protein. Interaction with nuclear receptors such as retinoid X receptor-α, for example, decreases retinoid X receptor-α—mediated transactivation activity (58). On the other hand, DEC1–STAT3 interaction enhances STAT3 phenotypes, pointing to increased transactivation of STAT3 (30). In this study, we have shown that knockdown of DEC1 decreases β-catenin and NFATc1, and the decrease is more profound in the nuclei (Fig. 8, *D* and *E*), suggesting that DEC1 is engaged in nuclear translocation process, although the precise mechanism remains to be determined.

The role of DEC1 established in this study regarding RA development is clinically of significance. It is well-known that some of RA symptoms such as stiffness are worse in the morning (2), and DEC1 is a clock gene, linking circadian rhythms to RA condition. In support of this connection, inflammatory cytokines such as TNF-α undergo circadian regulation in RA (3, 4). On the other hand, RA is closely related to osteopenia/osteoporosis condition. RA is a joint, but osteopenia/osteoporosis is a bone disease. Interestingly, our previous study has shown that DEC1 stimulates the PI3KCA/Akt/GSK3β signaling and prevents osteopenia/osteoporosis (28). In contrast, the current study has shown that DEC1 worsens RA condition, at least partially through activating the PI3KCA/Akt/GSK3β pathway (Figs. 6*A* and 8). The PI3KCA/Akt/GSK3β pathway has been increasingly used as a target for RA therapy (59, 60). With this regard, cautions must be exercised on the use of these therapeutics to prevent or minimize the development of osteopenia/osteoporosis.

In summary, our work points to several important conclusions. First, CIA, a widely used protocol for RA animal models, robustly induces RA phenotypes (*e.g.*, synovial hyperplasia) in DEC1^+/+^ but not DEC1^−/−^ mice, pointing to a strong engagement of DEC1 in the development of RA condition. Second, CIA greatly increases the expression of proinflammatory cytokines such as TNF-α in DEC1^+/+^ but not DEC1^−/−^ mice, and on the other hand, these very cytokines strongly induce DEC1, pointing to a DEC1-amplifying circuit for inflammation. Third, in addition to inflammatory signaling, DEC1 is shown to functionally interact with PI3KCA/Akt/GSK3β, Wnt/β-catenin, and NFATc1, pointing to an integral role DEC1 plays in developing rheumatoid condition. RA is one of the most common autoimmune diseases and affects almost 1% of the population. This study has provided important mechanistic understanding and laid a foundation of developing therapeutic strategies.

## Experimental procedures

### Chemical and reagents

Chicken type II collagen and complete Freund adjuvant were purchased from Chondrex. H&E staining kit was from Jiancheng Bio. Safranine red and Fast Green staining kit was from Solarbio. Dulbecco’s modified Eagle’s medium (DMEM) was from Gibco. Fetal bovine serum (FBS) was from Wisent. Diaminobenzidine peroxidase substrate kit was from Vector Laboratories. Transfection reagent GenJet was from SignaGen. Bicinchoninic acid protein assay kit was from Thermo Fisher. Enhanced chemiluminescence detection kit was from Vazyme. Cytokines such as TNF-α, IL-1β, IL-6, and LPS were from Peprotech. Nuclear and cytoplasmic extraction reagents were from Thermo Fisher. The antibody against DEC1 was described elsewhere (61). Anti-CTSK was from Santa Cruz Biotechnology. Anti-NFATc1 was from Cell Signaling Technology. All other antibodies were from BioGot. Human IL-6 ELISA Kit was from SHRBIO.

### Animals

**Fig. S1:**
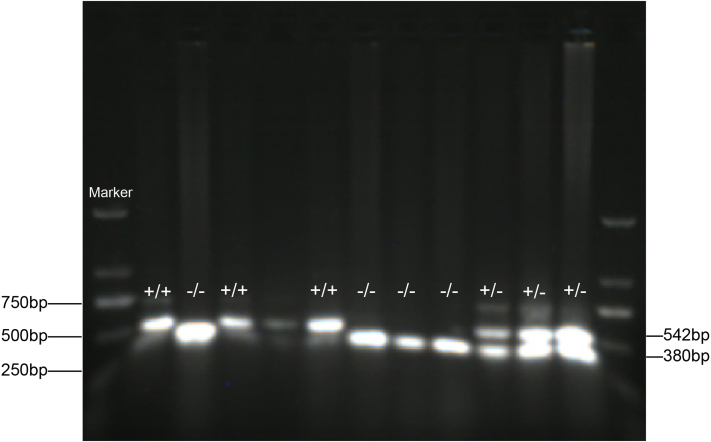


**Fig. S2:**
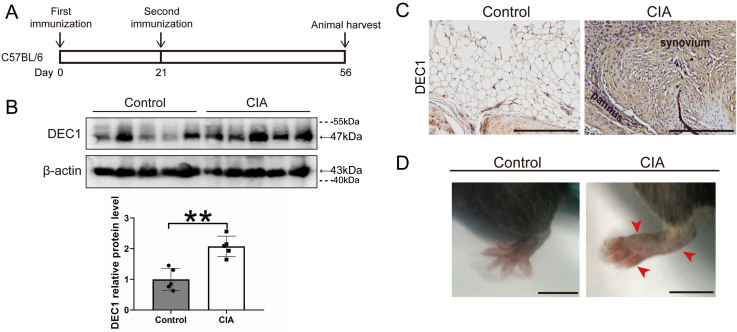


**Fig. S3:**
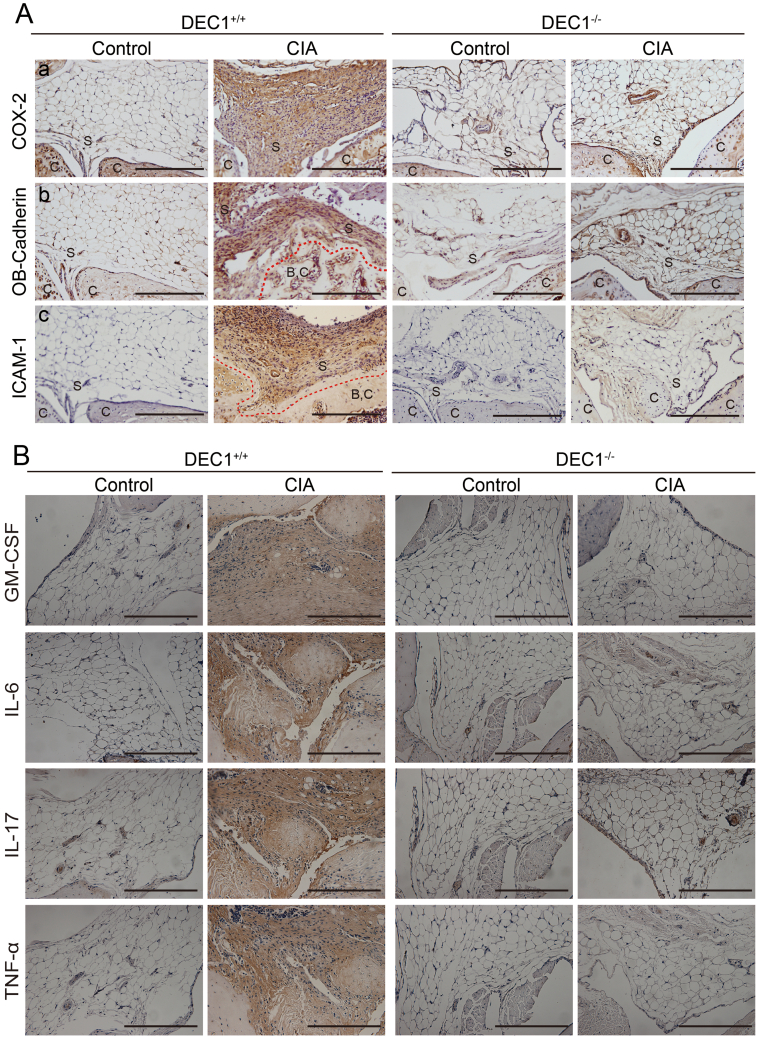


DEC1^+/−^ mice (RBRC04841) were obtained from RIKEN BioResource Center. Heterozygous female mice (DEC1^+/−^) were crossed with male counterparts to generate the WT (DEC1^+/+^) and DEC1 knockout (DEC1^−/−^) mice (24, 27). The chimeric mice were backcrossed to C57BL/6 for six to eight generations. In the breeding scheme, DEC1^+/+^ littermates were used as WT controls. The genotyping was performed right after birth and confirmed before the experiment to ensure the genotyping accuracy. Tail genetic identification is shown in Fig. S1. Mice had free access to food and water and were maintained in a pathogen-free animal facility under standard conditions (ambient temperature of 22 ± 2 °C, humidity of 55 ± 5%, and a 12 h:12 h light/dark cycle) in the Animal Core Facility of Nanjing Medical University. All animal experiments were conducted in accordance with the guidelines of the Institute for Laboratory Animal Research of Nanjing Medical University and approved by the Institutional Animal Care and Use Committee of Nanjing Medical University (IACUC-1903011).

### Collagen-induced RA model

Chicken type II collagen (2 mg/ml) was emulsified in complete Freund adjuvant (5 mg/ml *Mycobacterium tuberculosis*) to produce stable emulsion right before injections (days 0 and 21). DEC1^+/+^ and DEC1^−/−^ male mice (20 each genotype), at an age of 8 weeks, were injected intradermally at the base of the tail with 100 μl of an emulsified collagen (100 μg chicken type II collagen) on day 0 (initial immunization) and day 21 (booster immunization). Control mice (both DEC1^+/+^ and DEC1^−/−^ genotypes) were injected with the same volume (100 μl) of normal saline. Arthritis developed 8 weeks after the initial injection. Body weight, hind paw thickness, and arthritis score were monitored every 3 days starting on day 21 to day 56. Arthritis score was evaluated according to the manufacturer’s instruction. Mice were sacrificed on day 56 following anesthesia. Legs or protein samples were collected for further experiments.

### LiCl experiment

*In vivo*, WT, and age-matched DEC1 KO mice (8 weeks old) were subjected to RA induction by chicken type II collagen as described previously. RA mice were divided into three groups: DEC1^+/+^ CIA mice, DEC1^−/−^ CIA mice, and DEC1^−/−^ CIA mice treated with LiCl (five to six mice in each group).

The LiCl treatment group was intraperitoneally injected with LiCl at 200 mg/kg/day for 5 weeks starting on day 21 (booster immunization), as previously described (62), whereas mice in DEC1^+/+^ and DEC1^−/−^ groups were intraperitoneally injected with the same volume of PBS. Twenty-four hours after the last injection, mice were euthanized by chloral hydrate (400 mg/kg, intraperitoneally) and hind paw thickness and arthritis score were monitored. Legs and protein samples were collected for further experiments.

*In vitro*, MH7A cells transfected with vector or DEC1 siRNA plasmid were divided into three groups (vector, Si-DEC1, and Si-DEC1 + LiCl). The Si-DEC1 + LiCl group was treated with 5 mM LiCl (other two groups were treated with PBS) for 24 h. After that, the media were collected for content of IL-6, and cells were harvested for protein expression.

### Micro-CT analysis

Legs were fixed in 4% paraformaldehyde overnight and subjected to three-dimensional micro-CT analysis with a SkyScan scanner (SkyScan1172; Bruker, Inc). Micro-CT scanning was performed at the resolution of 18 μm. Trabecular parameters were evaluated including trabecular bone volume to total volume fraction, trabecular thickness (Tb.Th, μm) and trabecular number (Tb.N, 1/μm), and the separation between individual trabecular (Tb.sp, μm). The 3D bone mineral density (mg/cm^2^) was also determined. The 2D grayscale CT images were reconstructed in 1120 × 1120 pixel matrices by using Nreconver.1.6.1.5 (SkyScan), and 3D microstructural parameters were calculated (63).

### IHC and histological staining

The whole legs were fixed with 4% paraformaldehyde for 24 h and decalcified in EDTA buffer for 2 weeks. Thereafter, tissues were embedded in paraffin and sectioned at a thickness of 5 μm. The sections were dewaxed in xylene and rehydrated by decreasing ethanol solution. Rehydrated sections were treated with 3% H_2_O_2_ to quench endogenous peroxidase for 15 min and washed with PBS for three times. Sections were then infused in boiling 0.01 M citrate buffer retrieved antigen for 12 min to retrieve antigen and incubated with 10% goat serum at room temperature for 1 h to block none-specific binding sites. Thereafter, sections were incubated with primary antibodies, anti-DEC1 (1:1000 dilution), anti-COX-2 (1:200 dilution), anti-OB-cadherin (1:200 dilution), anti-ICAM-1 (1:400 dilution), anti-NFATc1 (1:200 dilution), anti-β-catenin (1:200 dilution), anti-PI3Kp110α (1:200 dilution), anti-MMP (1:200 dilution), anti-CTS (1:200 dilution), and anti-RANKL (1:200 dilution) overnight at 4 °C. The sections were washed with PBS for three times and incubated with the horseradish peroxidase–conjugated secondary antibodies at room temperature for 1 h. Sections were visualized by diaminobenzidine, and the immunoreactivity was detected with light microscope (BX53; Olympus). The negative control was incubated without primary antibody. Separately, sections were evaluated for synovitis, pannus, and bone erosion. Cartilage destruction was measured by Safranine red and Fast Green staining according to the manufacturer’s instruction.

### Cell culture and treatment

MH7A cell line, established from RA patient’s synovial fibroblast, was purchased from Guangzhou Jennio Biotech. Cells were cultured in DMEM supplemented with 10% FBS, penicillin (100 U/ml), and streptomycin (100 mg/ml) at 37 °C, 5% CO_2_, and 95% humidity. Cells were treated with TNF-α (0, 0.5, 1, 5, and 50 ng/ml), IL-1β (0, 0.2, 0.5, 2.5, and 25 ng/ml), IL-6 (0, 0.5, 1, 5, and 50 ng/ml), or LPS (0, 0.05, 0.1, 0.5, and 5 μg) for 24 and then harvested for further experiments.

### Knockdown of DEC1 with siRNA

MH7A cells were seeded overnight in 6-well plates at the density of 5 × 10^5^/well and then transfected with GeneJet (VerII) (SignaGen Laboratories). The transfection mixture contained 400 ng DEC1-siRNA or control-siRNA (Sigma–Aldrich). Cells were cultured in the transfection mixture for 6 h, and the medium was replaced with fresh regular medium and cultured for additional 16 h. For phosphorylated proteins, the cells (vector; Si-DEC1) were treated with TNF-α (5 ng/ml) for 1 and 2 h, the cells were harvested for detecting PI3Kp110α, p-Akt, p-GSK3β, β-catenin, and NFATc1 with Western blotting, the media were collected for IL-6 content with ELISA. For cytoplasmic and nuclear fractions, the transfected cells (vector, Si-DEC1) were harvested and prepared cytoplasmic and nuclear fractions as descripted later for detecting β-catenin and NFATc1 with Western blotting. TNF-α or LiCl was then administrated to the transfected cells for further studies.

### Western blotting

Tissues or cells were lysed by radioimmunoprecipitation assay buffer and centrifuged at 12,000 rpm for 15 min at 4 °C. Supernatants were collected and determined for protein concentrations by Bicinchoninic Acid Protein Assay Kit. Typically, protein (30 μg) was separated by 10% SDS-PAGE and electrophoretically transferred to polyvinylidene difluoride membrane by Bio-Rad Miniprotein-III wet transfer unit (Bio-Rad). The membrane was blocked by 5% nonfat milk or bovine serum albumin for 1 h at room temperature and incubated at 4 °C overnight with primary antibodies against DEC1 (1:2000 dilution), COX-2 (1:1000 dilution), OB-cadherin (1:1000 dilution), ICAM-1 (1:1000 dilution), MMP (1:1000 dilution), CTSL (1:1000 dilution), CTSK (1:1000 dilution), RANKL (1:1000 dilution), β-catenin (1:1000 dilution), NFATc1 (1:1000 dilution), PI3Kp110α (PI3KCA, 1:1000 dilution), p-Akt and Akt (1:1000 dilution), p-GSK3β and GSK3β (1:1000 dilution), β-actin (1:5000 dilution), histone H3 (1:1000 dilution), and GAPDH (1:3000 dilution). The membrane was washed for three times and incubated with horseradish peroxidase—conjugated secondary antibodies for 1 h at room temperature. The immunostaining was detected by enhanced chemiluminescence substrate, and the signal was captured by Image Analysis software (QinXiang Instrument). The relative protein level was normalized by the intensity of GAPDH or histone H3 staining.

### Extraction of nuclear and cytoplasmic fractions

Transfected cells (vector, Si-DEC1) were harvested and washed with ice-cold PBS for three times. Cytoplasmic and nuclear fractions were extracted with a nuclei isolation kit (Thermo Fisher). The nuclear and cytoplasmic fractions were analyzed by Western blotting for the expression of proteins of interest (β-catenin and NFATc1). The immunostaining of GAPDH was used as the normalization control for cytoplasmic fractions, whereas the immunostaining of histone H3 was used as the normalization control for nuclear fractions.

### Cell migration and invasion assays

Transfected MH7A cells (vector, Si-DEC1, and Si-DEC1 + LiCl) were seeded in the top chamber (8.0 μm) in DMEM at a density of 5 × 10^5^ for migration and 1 × 10^5^ for invasion, respectively. The bottom chamber was filled with medium containing 2.5% FBS for migration and 5% FBS for invasion. Cell migration was determined in the transwell format without matrix gel, but cell invasion was determined in the transwell with chambers being filled matrix gel. Cells were cultured at 37 °C, 5% CO_2,_ and 95% humidity for 12 h (migration) and 24 h (invasion). Cells were collected and fixed with 4% paraformaldehyde and subsequently stained with crystal violet. The media (vector, siDEC1, and siDEC1 + LiCl) were collected for IL-6 content with ELISA, and cell lysates were detected for OB-cadherin with Western blotting.

### ELISA

The media from transfected MH7A cells (vector, Si-DEC1, and Si-DEC1 + LiCl) were collected, and their concentrations of IL-6 were measured using Human IL-6 ELISA Kit (E6027H) according to the manufacturer’s protocol.

### Statistical analysis

Data are expressed as the mean ± SD. All data were repeated four times, and each group had at least five mice. The data were analyzed by one- or two-way ANOVA followed by Turkey’s or Sidak’s post hoc test (two-tailed), and paired comparisons were analyzed by *t* test (two-tailed). The analysis was conducted with the IBM SPSS Statistics software (version 25.0). Statistical significance was specified at the level of *p* < 0.05.

## Data availability

All data are contained within the article including in the section of supporting information.

## Supporting information

This article contains supporting information.

## Conflict of interest

The authors declare that they have no conflicts of interest with the contents of this article.

## Figures and Tables

**Fig. S4:**